# Homeless

**Published:** 1872-04

**Authors:** 


### HOMELESS,

And sick, and old, and poor. How dreadful! Yet any reader of these lines may become so before he dies. The man who by his money, and credit, and financial abilities saved the nation from bankruptcy in the Revolution, the learning of whose timely assistance brought tears to the eyes of the great Washington, died in prison in Philadelphia! Yes, the nation's saviour, financially, Robert Morris, languished in a debtor's jail. Many a man and woman who has rolled in wealth for a score or more of years, has died in abject poverty.

But a higher civilization is dawning; a god-like and a world-wide charity, a thoughtful generosity, is growing, and is spreading wider, and higher, and faster, all over Christendom, inspiring men and women of wealth to devise measures for taking care of the children of adversity; and it is showing itself in the direction of extensive buildings, to be made into " Homes " for various classes. There is a

"HOME FOR THE FRIENDLESS,"

a home for orphans, and now, in different parts of the land, there are homes in process of erection for old women who have seen better days. Among these is a

PRESBYTERIAN HOME

for the old, and sick, and infirm, founded by the liberality of a Presbyterian clergyman, and enlarged and endowed to a considerable extent by the beneficence of one of Philadelphia's merchant princes, Mr. Brown. The more money contributed, the more widely can the institution work. For the convenience of such as may desire to contribute something, which in the lapse of time is pretty certain to yield a return, either to the contributor or some descendant or relative or friend, the third article of the charter is here given :

"All the members of the Presbyterian Alliance, and all other persons who shall contribute to the Home the sum of thirty dollars or more, yearly, or the sum of five hundred dollars at one time, shall be members of this corporation, and be entitled to vote at the annual election of trustees."

Information will be cheerfully given, verbally or by letter, at "The Presbyterian Publication House," 1334 Chestnut Street, Philadelphia, Penn. Dr. Chapin's Church (Universalist), with a large liberality, is building a Home for its old and poor in New York; and it is to be hoped that every other denomination will follow the humane example set them, until the whole country shall be dotted with such

HAVENS OF REST, for the old and the sick of every class, and creed, and age, and sex, and condition ; and that is the noblest heart which resolves to do something in this direction, within the hour, it may be to the extent of a hundred thousand dollars, or dimes, or cents; nay, a single dollar may become the mustard-seed which shall become a tree in the future, to shelter thousands of humanity's unfortunates.

